# Stratifying the early radiologic trajectory in dyspneic patients with COVID-19 pneumonia

**DOI:** 10.1371/journal.pone.0259010

**Published:** 2021-10-22

**Authors:** Jin Young Kim, Keum Ji Jung, Seung-Jin Yoo, Soon Ho Yoon

**Affiliations:** 1 Department of Radiology, Dongsan Hospital, Keimyung University College of Medicine, Daegu, South Korea; 2 Department of Epidemiology and Health Promotion, Institute for Health Promotion, Graduate School of Public Health, Yonsei University, Seoul, South Korea; 3 Department of Radiology, Hanyang University Medical Center, Hanyang University College of Medicine, Seoul, South Korea; 4 Department of Radiology, UMass Memorial Medical Center, Worcester, MA, United States of America; 5 Department of Radiology, Seoul National University Hospital, Seoul, South Korea; Ohio State University Wexner Medical Center Department of Surgery, UNITED STATES

## Abstract

**Objective:**

This study aimed to stratify the early pneumonia trajectory on chest radiographs and compare patient characteristics in dyspneic patients with coronavirus disease 2019 (COVID-19).

**Materials and methods:**

We retrospectively included 139 COVID-19 patients with dyspnea (87 men, 62.7±16.3 years) and serial chest radiographs from January to September 2020. Radiographic pneumonia extent was quantified as a percentage using a previously-developed deep learning algorithm. A group-based trajectory model was used to categorize the pneumonia trajectory after symptom onset during hospitalization. Clinical findings, and outcomes were compared, and Cox regression was performed for survival analysis.

**Results:**

Radiographic pneumonia trajectories were categorized into four groups. Group 1 (n = 83, 59.7%) had negligible pneumonia, and group 2 (n = 29, 20.9%) had mild pneumonia. Group 3 (n = 13, 9.4%) and group 4 (n = 14, 10.1%) showed similar considerable pneumonia extents at baseline, but group 3 had decreasing pneumonia extent at 1–2 weeks, while group 4 had increasing pneumonia extent. Intensive care unit admission and mortality were significantly more frequent in groups 3 and 4 than in groups 1 and 2 (*P* < .05). Groups 3 and 4 shared similar clinical and laboratory findings, but thrombocytopenia (<150×10^3^/μL) was exclusively observed in group 4 (*P* = .016). When compared to groups 1 and 2, group 4 (hazard ratio, 63.3; 95% confidence interval, 7.9–504.9) had a two-fold higher risk for mortality than group 3 (hazard ratio, 31.2; 95% confidence interval, 3.5–280.2), and this elevated risk was maintained after adjusting confounders.

**Conclusion:**

Monitoring the early radiologic trajectory beyond baseline further prognosticated at-risk COVID-19 patients, who potentially had thrombo-inflammatory responses.

## Introduction

Coronavirus disease 2019 (COVID-19) is a contagious respiratory infection that was first identified in December 2019 in Wuhan, China, and then spread rapidly worldwide [1]. As of February 2021, more than 100 million people have been diagnosed with COVID-19, and over 2 million people have died of the condition [2]. The disease course of COVID-19 varies from asymptomatic to fatal, and one-fifth of patients with COVID-19 experience moderate to severe disease, especially those who are older and have comorbidities [3].

Chest radiography is an easily accessible imaging tool to assess and monitor pneumonia. COVID-19 pneumonia typically shows bilateral peripheral ground-glass opacities with or without consolidation [4]. The radiologic extent of pneumonia shows a dynamic change within 2 weeks after symptom onset; generally, its extent increases within 10 days, followed by a gradual decrease [5]. The trajectory of these early dynamic changes varies across patients with COVID-19. In some patients, the initial pneumonia extent remains similar or improves during the disease course [6], whereas pneumonia deteriorates rapidly after a few days in other patients, even if they show mild pneumonia at first [7].

The Fleischner Society and World Health Organization recommended using chest imaging in patients with moderate to severe disease and those at risk for disease progression [8, 9]. Dyspnea demanding hospitalization is a representative indicator for moderate to severe disease and a salient risk factor for progression in COVID-19 [10]. Considering the variation in the dynamic changes of pneumonia during the early disease course of COVID-19, analyzing the trajectory of pneumonia on chest radiographs will expand our understanding of the role of radiologic monitoring for managing vulnerable populations. This study aimed to stratify the early trajectory of pneumonia extent on chest radiographs and to compare the characteristics of dyspneic patients with COVID-19 according to the radiologic pneumonia trajectory.

## Materials and methods

Our study was approved by institutional review boards of Keimyung University Dongsan Hospital (2020-09-072) and Seoul National University Hospital (2010-022-1161). The requirement for patients to provide informed consent was waived.

### Patients

We retrospectively reviewed admitted COVID-19 patients from January through September 2020 at two hospitals in South Korea. This study included consecutive COVID-19 patients who were hospitalized due to dyspnea and had chest radiographs taken. Since this study focused on the course of chest radiographs during hospitalization, we excluded patients who did not receive chest radiographs within the first 2 weeks after symptom onset or who had only one chest radiograph. All patients were diagnosed as having COVID-19 based on a positive real-time polymerase chain reactive assay for severe acute respiratory syndrome coronavirus-2.

**Fig 1:**
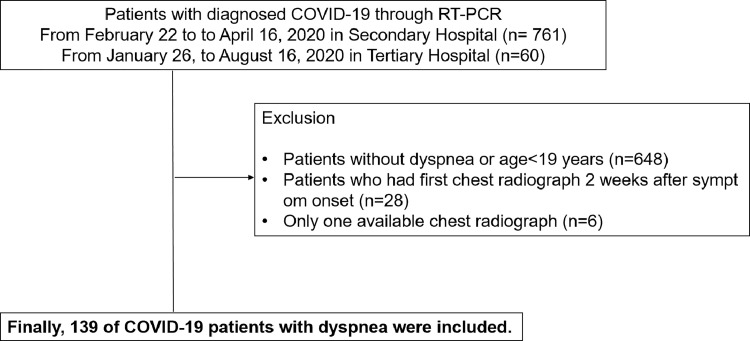
Patient flow diagram. COVID-19 = coronavirus disease 2019, n = number of patients, RT-PCR = real time polymerase chain reaction.

Among 173 dyspneic patients with COVID-19 (147 patients from a secondary hospital and 26 patients from a tertiary hospital) who had chest radiographs taken, 34 patients were excluded because 28 patients had their first chest radiographs 2 weeks after symptom onset and six patients had only one available chest radiograph. Finally, a total of 139 patients (114 and 25 patients from the two hospitals, respectively) were included in this study (Fig 1).

Of the 147 patients from a secondary hospital, 19 and 18 patients were overlapped with previous studies, respectively [7, 11]. Fifteen of the 147 patients and 3 of the 26 patients from a tertiary hospital were included in a COVID-19 imaging cohort [12].

### Data extraction

We reviewed patients’ clinical data as follows: 1) basic clinical information: age, sex, underlying diseases (e.g., hypertension, diabetes, cardiovascular disease, or cancer), COVID-19 diagnosis date, symptom type, and onset date; 2) initial and follow-up laboratory findings: white blood cell count (WBC) including absolute neutrophil count and absolute lymphocyte count, platelet count, C-reactive protein level (CRP), and lactate dehydrogenase (LDH); Follow-up laboratory results were extracted at four intervals: 3–5 days, 6–7 days, 8–10 days, and 11–14 days after admission; 3) clinical outcomes: whether patients had received oxygen supplementation, were admitted to the intensive care unit (ICU), received mechanical ventilation or extracorporeal membrane oxygenation (ECMO), or died due to COVID-19 deterioration.

### Quantifying pneumonia extent on chest radiographs

Pneumonia extent on chest radiographs was automatically assessed using a deep learning algorithm. Anonymized DICOM files of chest radiographs were uploaded to a web-based deep learning quantification tool (http://tisepx.com/; TiSepX COVID-19, MEDICALIP), and the tool calculated total lung area, COVID-19 pneumonia area, and the proportion of pneumonia (%) on radiographs within a few seconds. The tool was based on the generative adversarial network that was trained by 50,000 consecutive images (submitted). The network-driven radiographic pneumonia extent underestimated the CT-driven pneumonia extent by 6.1% on average (95% limits of agreement: -29.5% to 17.2%), with a Pearson correlation coefficient of 0.748 in four external validation datasets. We categorized days after symptom onset into 11 intervals (0–3 days, 4–6 days, 7–9 days, 10–12 days, 13–15 days, 16–18 days, 19–21 days, 22–24 days, 25–27 days, 28–30 days, more than 30 days) and the maximum extent of pneumonia on a chest radiograph in each interval was used for analysis [13].

### Stratifying radiographic trajectory

Our study was analyzed by a statistician who experienced group-based modeling [14]. A group-based trajectory model using nonparametric maximum likelihood estimation was performed to identify clear trajectories of the proportion of pneumonia (%) changes during hospitalization [15, 16]. In this study, latent class growth modeling, a type of group-based trajectory model, was used. This analysis identified a relatively homogeneous cluster of trajectories of change over time in repeated observations of the subjects of analysis using multidimensional mixed modeling strategies [17]. The optimal fit model was determined by the Bayesian information criterion scores and 95% confidence intervals, assuming linear, quadratic, and tertiary patterns of variation of the proportion of pneumonia (%) changes.

We estimated the appropriate trajectory model by increasing the number of clusters from three to five. The best-fit model was chosen by considering the Bayesian information criterion index and clinical characteristics for each group. Each patient was assigned to the class with the highest probability. To ensure that all classes acquired had a clinically significant size, a condition was set that each class should contain at least 5% of the patients. Trajectory analyses were conducted based on the 11 intervals mentioned above, using the PROC TRAJ command in SAS (SAS Institute, Inc., Cary, NC, USA), which is used for group-based modeling of longitudinal data [16].

### Statistical analysis

Categorical variables are presented as numbers with percentages, and continuous variables are presented as means and standard deviations or median and interquartile ranges. Differences between groups were analyzed using the chi-square or Fisher exact tests for categorical variables and the independent Student *t-*test or Mann Whitney *U* test for continuous variables. Cox regression analyses were performed to predict ICU admission and mortality according to groups, and groups 1 and 2 were combined to serve as a reference because ICU admission and mortality did not occur in group 1. *P* values below 0.05 were considered to indicate statistical significance. Statistical analyses were performed using SPSS version 25.0 (IBM Corp., Armonk, New York, USA) and SAS version 9.4 (SAS Institute, Inc., Cary, NC, USA).

## Results

### Four different radiologic trajectory groups

**Fig 2:**
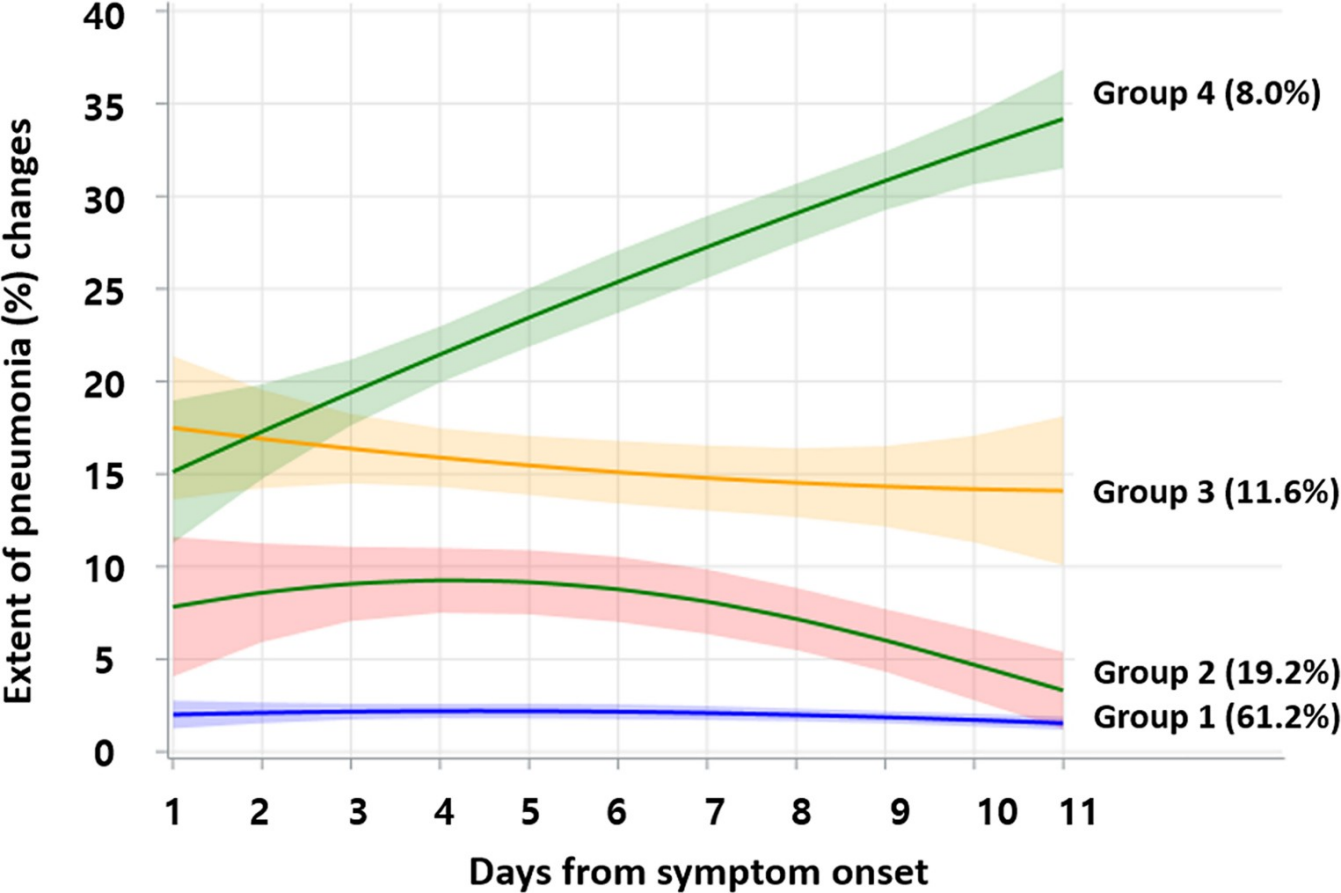
Extent of pneumonia (%) changes on chest radiographs by trajectory. There were four different radiologic trajectories. Group 1 (blue) showed a minimal extent of pneumonia during the total hospitalization period. Group 2 (pink) showed initial pneumonia at the time of diagnosis and gradually recovered. Group 3 (yellow) and group 4 (green) showed a similar mild extent of pneumonia at the time of admission, but group 3 showed a recovering course, while group 4 showed a worsening course. *Days after symptom onset: 1, 0–3 days; 2, 4–6 days; 3, 7–9 days; 4, 10–12 days; 5, 13–15 days; 6, 16–18 days; 7, 19–21 days; 8, 22–24 days; 9, 25–27 days; 10, 28–30 days; 11 31 days or later.

**Fig 3:**
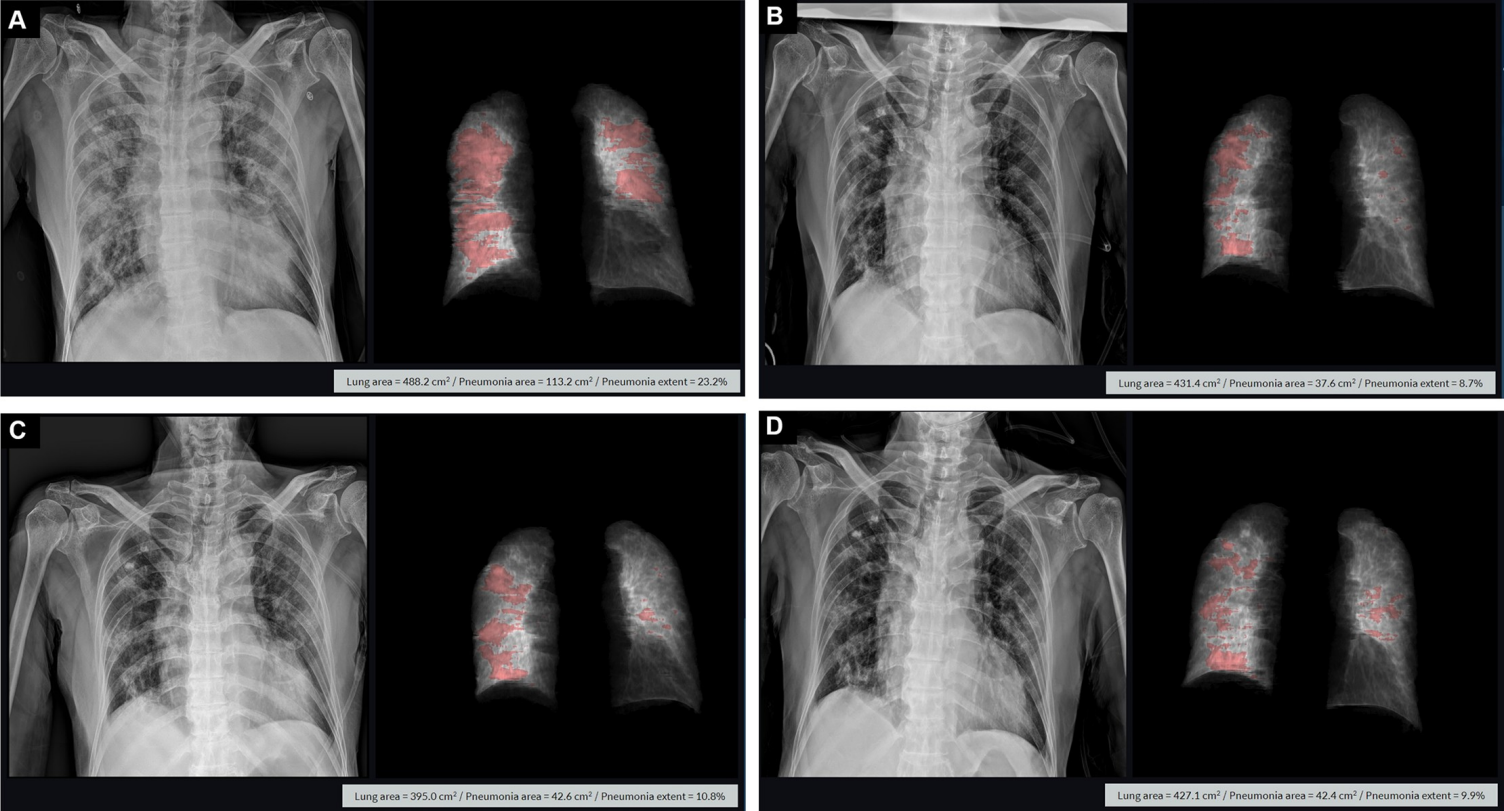
Chest radiograph course of a patient in group 3. A 78-year-old male COVID-19 patient showed a 23% extent of pneumonia on an initial chest radiograph (A) taken on day 7 after symptom onset. On chest radiographs taken at 11 days (B), 14 days (C), and 17 days (D) after symptom onset, pneumonia extent decreased to 8.7%, 10.8% and 9.9% respectively. The patient received oxygen supplementation through a mask and antiviral treatment in the general ward and was normally discharged.

**Fig 4:**
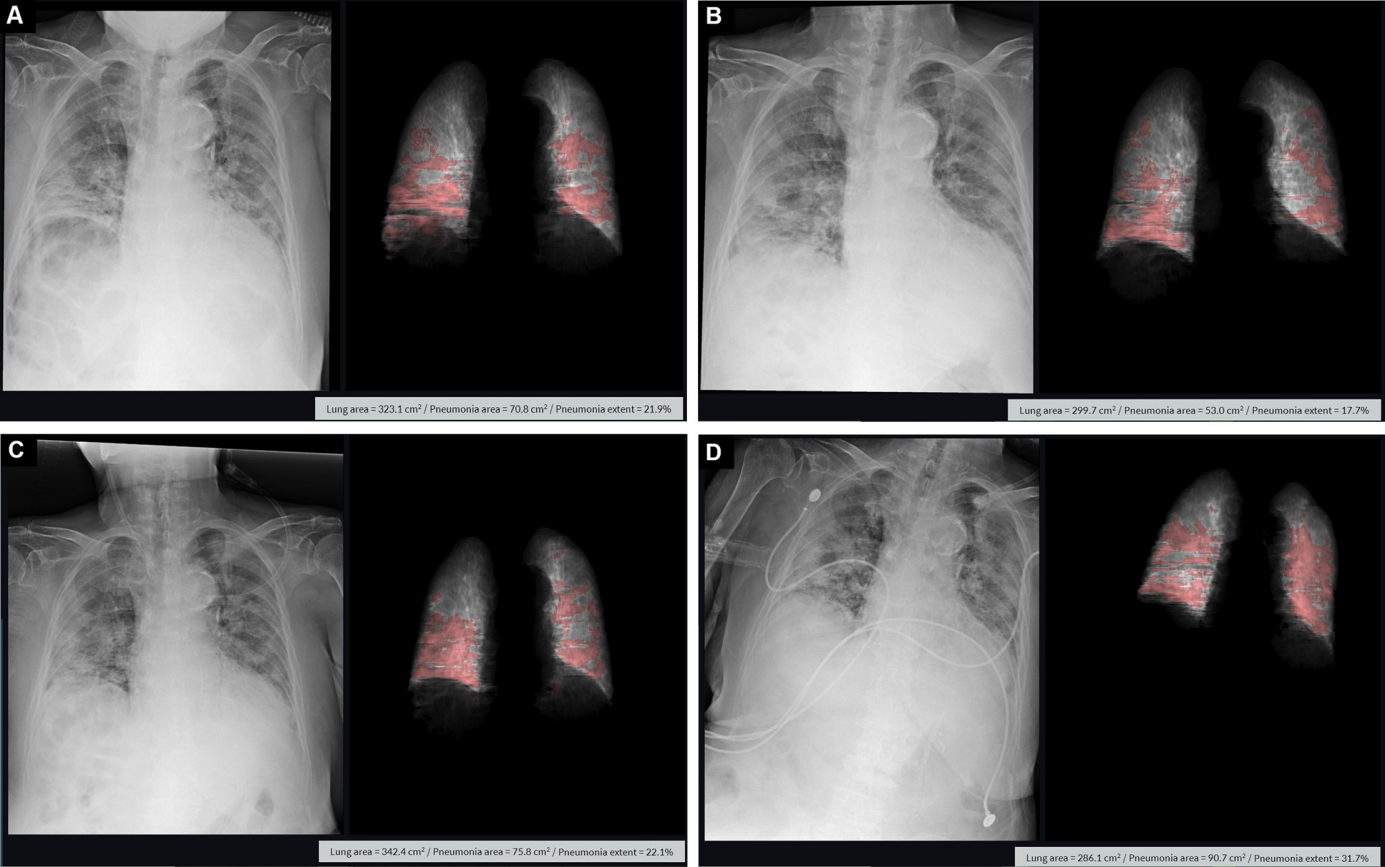
Chest radiograph course of a patient in group 4. A 92-year-old female COVID-19 patient showed a 21.9% extent of pneumonia on an initial chest radiograph (A) taken on the second day after symptom onset. On chest radiographs taken 6 days (B), 9 days (C) and 11 days (D) after symptom onset, pneumonia extent gradually increased to 17.7%, 22.1% and 31.7% respectively. The patient died from aggravated COVID-19 pneumonia.

The radiographic trajectories of the study population were stratified into four groups (Fig 2): group 1, 83 patients (59.7%); group 2, 29 patients (20.9%); group 3, 13 patients (9.4%); and group 4, 14 patients (10.1%). Group 1 showed negligible pneumonia on radiographs at baseline (pneumonia extent, 0.9%; 95% confidence interval [CI], 0.1%-1.8%) and during follow-up. Group 2 showed mild pneumonia at baseline (pneumonia extent, 7.6%; 95% CI, 1.9%-13.3%), which did not worsen much and gradually improved during follow-up. At baseline, groups 3 and 4 showed a similar extent of pneumonia (group 3, 16.9%; 95% CI: 5.0%-28.8%; group 4, 13.9%; 95% CI, 5.3%-22.4%) (Fig 3). However, the extent of pneumonia in group 4 gradually increased, while that in group 3 decreased. The difference between groups 3 and 4 became significant 2 weeks after symptom onset (group 3, 16.8% [95% CI, 10.8%-22.7%] versus group 4, 26.5% [95% CI, 20.2%-32.8%], *P* = .020) (Fig 4).

### Patient characteristics categorized according to baseline pneumonia extent

Table 1 summarizes patient characteristics according to baseline pneumonia extent. As groups 3 and 4 were not distinct in terms of pneumonia extent at baseline, the groups are presented combined. Group 1 was significantly younger than the other groups (*P* < .05), and the oldest patients were found in group 4. However, there was no statistically significant difference in age among groups 2 to 4 (post-hoc analysis, all *P* > .05). Sore throat and gastrointestinal symptoms were significantly more common in groups 1 and 2 than in other groups (both *P* < .05). Other symptoms were similarly distributed across groups (all, *P* >.05). Group 1 had fewer comorbidities, including hypertension, diabetes, and cardiovascular disease, than groups 2 to 4 (*P* < .05). Initial WBC, CRP, and LDH were significantly lower, and the absolute lymphocyte count was significantly higher in group 1 than in groups 2 to 4 (all, *P* < .05). ICU admissions and death were significantly more common in groups 3 and 4 than in groups 1 and 2 (both *P* < .05).

**Table 1:** Patient characteristics categorized according to baseline pneumonia extent.

Parameters	Total (n = 139)	Group 1 (n = 83)	Group 2 (n = 29)	Group 3 and 4 (n = 27)	P value
**Age**	62.7±16.3	55.4±14.8	69.4±14.1	77.8±7.2	<0.001
**Sex (M/F)**	52/87	21(25.3)/62 (74.7)	17 (58.6)/12 (41.4)	14 (51.9)/13(48.1)	0.001
**Symptom type**					
**Fever**	88 (63.3)	48 (57.8)	21 (72.4)	19 (70.4)	0.261
**Cough**	95 (68.3)	57 (68.7)	20 (69.0)	18 (66.7)	0.978
**Sputum**	83 (59.7)	48 (57.8)	20 (69.0)	15 (55.6)	0.510
**Myalgia**	66 (47.5)	45 (54.2)	13 (44.8)	8 (29.6)	0.080
**Sore throat**	47 (33.8)	37 (44.6)	5 (17.2)	5 (18.5)	0.005
**Gastrointestinal symptom**	43 (30.9)	29 (34.9)	11 (37.9)	3 (11.1)	0.044
**Underlying disease**					
**Hypertension**	51 (36.7)	19 (22.9)	16 (55.2)	16 (59.3)	<0.001
**Diabetes**	32 (23.0)	10 (12.0)	12 (41.4)	10 (37.0)	0.001
**Cardiovascular disease**	11 (7.9)	3 (3.6)	7 (24.1)	1 (3.7)	<0.001
**Cancer**	6 (4.3)	5 (6.0)	0 (0)	1 (3.7)	0.810
**Initial SpO2 (%)** *	96 (94–98)	97 (95–98)	95 (93–96.5)	91 (85.8–95.0)	
**Initial laboratory findings**					
**WBC (10**^**3**^**/uL)**	5.160 (4.120–7.500)	4.580 (3.980–6.130)	5.580 (4.720–8.315)	7.940 (3.940–11.220)	0.001
**Absolute neutrophil count (/uL)**	3240.0 (2314.0–488.50)	2755.6 (2000.0–3820.0)	3850.0 (3334.5–6110.0)	5800 (2800–8628)	<0.001
**Absolute lymphocyte count (/uL)**	1285.0 (930.0–1720.0)	1520.0 (1220.0–1830.0)	1078.5 (831.2–1341.4)	960.0 (580.0–1210.0)	<0.001
**Platelet (10**^**3**^**/uL)**	216.5 (176.0–276.0)	220.0 (178.0–279.0)	202.0 (144.5–295.0)	203.0 (154–237.0)	0.190
**CRP (mg/dL)**	2.45 (0.3–8.0)	0.7 (0.1–2.1)	8.3 (5.7–13.8)	11.4 (5.3–14.6)	<0.001
**LDH (U/L)†**	503.0 (401.0–719.5)	466.0 (397.0–580.0)	538.0 (348.5–695.0)	928 (705.0–1264.0)	<0.001
**Treatment**					
**Oxygen supplement (nasal or mask)**	73 (52.5)	23 (79.3)	23 (79.3)	27 (100)	<0.001
**Mechanical ventilation**	17 (12.2)	0 (10.3)	3 (38.5)	14 (51.9)	<0.001
**ECMO**	3 (2.2)	0 (0)	0 (0)	3 (11.1)	0.007
**Outcome**					
**ICU admission**	18 (12.9)	0 (0)	3 (10.3)	14 (51.9)	<0.001
**Death**	14 (10.2)	0 (0)	1 (3.6)	13 (50)	<0.001

Data are present as number of patients (percentages) or median (interquartile ranges)

CRP, C-reactive protein; ECMO, extracorporeal membrane oxygenation; ICU, intensive care unit; LDH, lactate dehydrogenase; SpO2, saturation of percutaneous oxygen; WBC, white blood counts

*Eleven patients (10 patients in group 1, 1 patient in group 3) did not have initial SPO2 data.

† Eight patients did not have initial LDH data (1 patient in group 1, 4 patients in group 2, 1 patients in group 3, 2 patients in group 4).

### Differences in characteristics between group 3 and 4 based on radiologic follow-up

**Fig 5:**
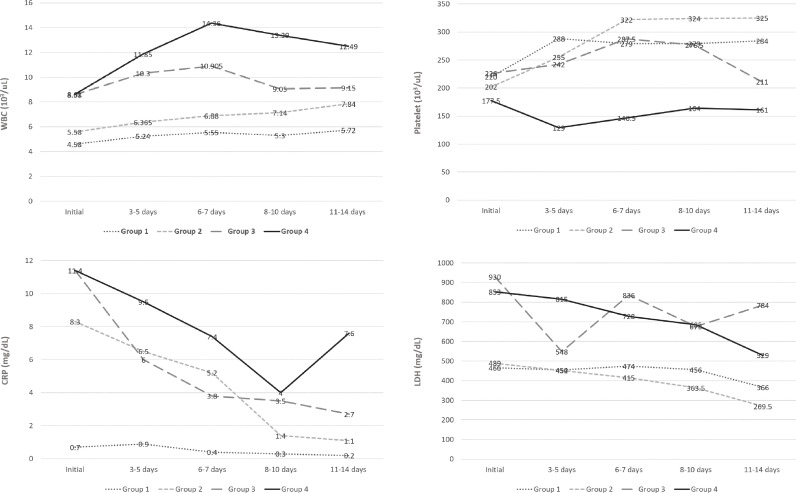
Laboratory findings during the course of hospitalization by trajectory. (A) White blood cell count. (B) Platelet count. (C) C-reactive protein. (D) Lactate Dehydrogenase.

Table 2 shows baseline characteristics, and Fig 5 shows trends of laboratory findings in groups 3 and 4. Initial thrombocytopenia (<150×10^3^/μL) was significantly more frequent in group 4 than in group 3 (*P* = 0.016). However, other known adverse inflammatory markers such as lymphopenia (<1000/μL), neutrophilia (>7700/μL), high CRP (>10 mg/dL), and high LDH (>450 U/L) did not significantly differ between group 3 and 4 (all, *P* >.05). During hospitalization, platelet counts remained lower in group 4 than in group 3 and significantly differed at 3–5 days and 6–7 days after admission (both, *P* < .05) (Fig 5). However, other laboratory findings, including WBC, CRP, and LDH, were not significantly different during hospitalization (all, *P* >.05).

**Table 2:** Characteristics between group 3 and group 4 as differentiated during radiologic follow-up.

Parameters	Group 3 (n = 13)	Group 4 (n = 14)	P value
**Age**	75.9±7.5	79.6±6.6	0.191
**Sex (M/F)**	7 (53.8)/6 (46.2)	7 (50)/7 (50)	0.842
**Symptom type**			
**Fever**	8 (61.5)	11 (78.6)	0.420
**Cough**	7 (53.8)	11 (78.6)	0.236
**Sputum**	6 (46.2)	9 (64.3)	0.449
**Myalgia**	3 (23.1)	5 (35.7)	0.678
**Sore throat**	1 (7.7)	4 (28.6)	0.326
**Underlying disease**			
**Hypertension**	8 (61.5)	8 (57.1)	0.816
**Diabetes**	6 (46.2)	4 (28.6)	0.440
**Cardiovascular disease**	0 (0)	1 (7.1)	>0.999
**Cancer**	1 (7.7)	0 (0)	0.481
**Initial SpO2<93%**	5 (41.7)	10 (71.4)	0.126
**Laboratory findings**			
**Initial WBC**	8.580 (6.325–9.345)	8.610 (3.480–15.008)	0.720
**Absolute neutrophil count>7700uL**	2 (15.4)	6 (42.9)	0.209
**Absolute lymphocyte count<1000uL**	5 (38.5)	9 (64.3)	0.180
Platelet <150 *10^**3**^**/uL**	0 (0)	6 (42.9)	0.016
**CRP>10mg/dL**	8 (61.5)	7 (50.0)	0.547
**LDH>450U/L**	10 (83.3)	12 (100)	0.239
**Treatment**			
**Oxygen supplement (nasal or mask)**	13 (100)	14 (100)	0.257
**Mechanical ventilation**	5 (38.5)	9 (64.3)	0.180
**ECMO**	0 (0)	3 (21.4)	0.222
**Outcome**			
**ICU admission**	6 (46.2)	8 (57.1)	0.568
**Death**	4 (33.3)	9 (64.3)	0.116

Data are present as number of patients (percentages). CRP, C-reactive protein; ECMO, extracorporeal membrane oxygenation; ICU, intensive care unit; LDH, lactate dehydrogenase; SpO2, saturation of percutaneous oxygen; WBC, white blood counts

### Association of radiologic trajectory and clinical outcomes

Table 3 shows the association of radiologic trajectory of groups 3 and 4 with ICU admission and mortality. Group 4 had two-fold higher hazard ratios (HRs) for mortality than group 3 when groups 1 and 2 served as a reference. Univariate analyses (model 1) revealed that groups 3 and 4 were significantly associated with ICU admission (HR in group 3, 33.8 [95% CI, 6.79–168.35]; and HR in group 4, 39.43 [95% CI, 8.33–186.66]) and mortality (HR in group 3, 31.18 [95% CI 3.47–280.19]; and HR in group 4, 63.28 [95% CI, 7.93–504.90]). When adjusting for age and sex (model 2) or age, sex, and diabetes (model 3), groups 3 and 4 continued to have statistically significant associations with both outcomes:

For ICU admission, in model 2, the HR in group 3 was 23.39 (95% CI, 4.10–133.33) and the HR in group 4 was 24.75 (95% CI, 4.21–145.53). In model 3, for ICU admission, the HR in group 3 was 21.39 (95% CI, 3.53–129.76) and the HR in group 4 was 26.77, (95% CI, 4.49–159.2). For mortality, in model 2, the HR in group 3 was 24.1 [95% CI, 2.29–255.94] and the HR in group 4 was 44.20 (95% CI, 4.20–464.7). In model 3, for mortality, the HR in group 3 was 19.87 (95% CI, 1.75–225.63) and the HR in group 4 was 38.06 (95% CI, 83.95–366.71).

**Table 3:** Hazard ratios for mortality and ICU admission according to groups stratified by radiographic trajectory.

		Trajectory Group of total lung area, COVID-19 pneumonia area and the proportion of pneumonia (%) changes		
		Group 1 and 2	Group 3	Group 4
N		112	13	14
Death				
Event		1	4	9
HR (95% CI)	Model 1	1.0	31.18 (3.47–280.19)	63.28 (7.93–504.90)
	Model 2	1.0	24.10 (2.29-255-94)	44.20 (4.20–464.78)
	Model 3	1.0	19.87 (1.75–225.63)	38.06 (3.95–366.71)
ICU admission				
Event		3	6	8
HR (95% CI)	Model 1	1.0	33.80 (6.79–168.35)	39.43 (8.33–186.66)
	Model 2	1.0	23.39 (4.10–133.33)	24.75 (4.21–145.53)
	Model 3	1.0	21.39 (3.53–129.76)	26.77 (4.49–159.52)

Model 1: crude model; Model 2: adjusted for age and sex; Model 3: adjusted for age, sex and diabetes mellitus. CI, confidence interval; ICU, intensive care unit; HR, hazard ratio

## Discussion

This study stratified dyspneic patients with coronavirus disease 2019 (COVID-19) into four groups depending on the radiographic trajectory of pneumonia extent. One-fifth of the patients (group 3, 9.4%; group 4, 10.1%) presented with pneumonia involving around 15% of the lungs at symptom onset, while four-fifths of the patients had negligible (group 1, 59.7%) to mild pneumonia (group 2, 20.9%). This patient proportion seems to fit the well-known finding that 20% of patients with COVID-19 suffer from critical disease, whereas the others have a mild disease course. The patients in groups 3 and 4 tended to be older, had more frequent comorbidities or laboratory abnormalities, and were more likely to experience intensive care unit admission and mortality. These findings are in accordance with prior observations of patients with a larger extent of COVID-19 pneumonia based on baseline radiologic examinations [18–20].

Monitoring the trajectory of pneumonia extent further separated dyspneic patients who had a similar initial pneumonia extent and could not be differentiated at baseline: pneumonia improved in group 3, whereas pneumonia became aggravated in group 4. A statistical comparison of baseline characteristics between the two groups was somewhat limited due to the small number of patients. There were non-significant trends for older age, higher absolute neutrophil count, lower absolute lymphocyte count, more frequent mechanical ventilation, and higher risk of mortality in group 4, but most of the clinical characteristics were not statistically different at baseline except for thrombocytopenia. Thrombocytopenia exclusively occurred and persisted during hospitalization in group 4 patients, along with an increasing trend of inflammatory markers during hospitalization (Fig 5). Thrombocytopenia, typically mild degree, is prevalent in severe cases of COVID-19 [21] and is considered to be the most sensitive marker of sepsis-induced coagulopathy [22]. Platelets are at the frontline of pathogenic inflammation and thrombosis in COVID-19, which is referred to as thrombo-inflammation [23], and thrombocytopenia is a predictor of mortality [24, 25] Therefore, it is possible that the patients in group 4 may have been at a higher risk of thrombo-inflammatory responses to COVID-19 than those in the other groups.

Interestingly, the HRs for mortality were twice as high in group 4 than in group 3 regardless of adjustment, while both groups had similar HRs for ICU admission. The higher mortality in group 4 might be partly explainable by coagulopathy, considering that coagulopathy has consistently been reported as a major risk factor for mortality in COVID-19 [25, 26]. The radiologic trajectories of pneumonia extent between groups 3 and 4 began to diverge 1 week after symptom onset, and the difference between the groups became clearly noticeable 2 weeks after symptom onset. As a considerable portion of thrombotic complications and mortality occur 1–2 weeks after symptom onset [27, 28], monitoring the early radiographic trajectory of pneumonia might enable early identification of the prothrombotic phenotype in patients with COVID-19.

We believe that aggravating or improving trends of pneumonia on chest radiographs can be monitored in various ways, including a visual or semi-quantitative assessment by experienced radiologists or automatic quantification with the support of up-to-date technologies. This study adopted a fully-automated neural network providing pneumonia extent (%) as a continuous variable to apply a group-based trajectory model for categorization. Chest radiography is the most commonly used type of imaging examination and is accessible even in a resource-limited setting, but has a wide variation in imaging acquisition procedures. Readings by radiologists with COVID-19 experience would be a realistic and readily applicable way to differentiate radiographic trajectories in most facilities in light of variation in acquisition.

There are several limitations of this study. First, the study population was relatively small, and it had a retrospective design. Second, there were no additional coagulation data other than platelets such as D-dimer, prothrombin time, fibrinogen, or fibrin degradation product. Our observations warrant an investigation in a large population with detailed coagulation tests. Third, since all patients did not receive chest radiographs at regular intervals, there were some missing data in each interval. Fourth, the 95% CIs of HRs for ICU admission or mortality were wide because these adverse outcomes were scarce in the reference group. Fifth, the radiographic quantification of pneumonia extent reflects an underestimation relative to quantification based on chest CT scans and could be affected by the level of inspiration or position during radiographic examinations.

## Conclusion

In conclusion, monitoring the early trajectory of pneumonia extent on chest radiographs could further stratify patients at risk for worse outcomes beyond baseline examinations. Patients with aggravated coronavirus disease 2019 pneumonia on radiographs were prone to have thrombocytopenia and mortality. Those patients might have thrombo-inflammatory abnormalities, and radiographic monitoring might help identify this at-risk population.

## Abbreviations

CI: Confidence interval
COVID-19: Coronavirus disease 2019
CRP: C-reactive protein
HR: Hazard ratio
ICU: Intensive care unit
LDH: Lactate dehydrogenase
WBC: White blood cell
